# Orbital Magnetic Resonance Imaging May Contribute to the Diagnosis of Optic Nerve Lymphoma

**DOI:** 10.3389/fneur.2020.00301

**Published:** 2020-04-28

**Authors:** Mo Yang, Jie Zhao, Honglu Song, Shihui Wei, Huanfen Zhou, Quangang Xu

**Affiliations:** ^1^Department of Ophthalmology, The First Medical Center, Chinese PLA General Hospital, Beijing, China; ^2^Department of Ophthalmology, The 980th Hospital of the Chinese PLA Joint Logistics Support Force, Shijiazhuang, China

**Keywords:** optic nerve, lymphoma, magnetic resonance imaging, cerebrospinal fluid, central nervous system

## Abstract

**Background:** Optic nerve lymphoma can present a diagnostic challenge because of its confusing clinical features and the difficulty of obtaining lesion tissue for biopsy. The objective of this study was to find some flags of lymphomatous infiltration of optic nerves.

**Methods:** We report two cases of optic nerve lymphoma and conduct a literature review to determine whether a common diagnostic characteristic can be identified.

**Results:** We examined 22 optic nerve lymphoma cases. Thirteen cases were systemic lymphoma infiltration of the optic nerve, five were primary central nervous system lymphoma (PCNSL), and four were primary isolated optic nerve lymphoma. Twenty patients manifested significant enlargement of the lesions in orbital/brain MRI. Seventeen contrast-enhanced MRIs showed abnormal enhancement of the optic nerve. All PCNSL and isolated optic nerve lymphoma patients in the series showed marked enhancement. Moderate and subtle enhancement was found in systemic lymphoma patients only. At the enhancement site, six isolated optic nerve lymphoma and PCNSL patients presented intrinsic enhancement, ten systemic patients showed both optic nerve and sheath enhancement, and one demonstrated sheath enhancement. Cerebrospinal fluid (CSF) tests showed elevated protein levels in six patients, and a neoplasm in one patient. We found abnormality of CSF immunity in both of our patients.

**Conclusion:** Combined characteristics of orbital MRI and CSF tests may facilitate expeditious suspicion establishment of optic nerve lymphoma.

## Introduction

Lymphoma can involve any organ in the body and presents with a wide range of symptoms (1). Visual impairments can be the chief complaint. Most cases are characterized by ocular involvement; optic nerve infiltration only is a rare condition.

Sporadic case reports on optic nerve involvement as the initial or secondary manifestation of primary central nervous system lymphoma (PCNSL) or metastatic lymphoma have been published. According to these reports, optic nerve lymphoma can be confused with a variety of inflammatory and neoplastic infiltrations of the optic nerve in clinical and radiographic examinations. Although in several case reports, optic nerve lymphoma was successfully diagnosed by optic nerve biopsy (2–4), biopsies are challenging due to the considerable risk of loss of visual acuity (2) and the difficulty of obtaining enough lesion tissue. Therefore, identifying some clinical characteristics of optic nerve lymphoma is essential to differentiate them from other optic nerve diseases. The aim of our study was to describe magnetic resonance imaging (MRI) and clinical characteristics in two optic nerve lymphoma patients admitted to the neuro-ophthalmology department of the People's Liberation Army General Hospital (PLAGH; Beijing, China) and to review the literature of optic nerve lymphoma in order to find some flags of lymphomatous infiltration of the optic nerves.

## Cases Reports

### Case 1

A 55-year-old man was referred to the neuro-ophthalmology clinic in April 2015, complaining about progressive, painless loss of vision in both eyes for 4 months. He reported no headache, weight loss, night sweats, or other neurological deficits.

Mild optic oedema had been found in his right eye on his first visit to an ophthalmologist 4 months previously. He was diagnosed with optic neuritis and received a treatment of oral prednisone (50 mg once daily) and triamcinolone injection around his right eyeball (40 mg, once). His vision improved after those treatments but subsequently deteriorated. Blindness had developed in his left eye about 1.5 months before his admission to the PLAGH. Deteriorated vision in his right eye had also been reported 2 days previously. His visual acuity (VA) at presentation was 0.02 (OD), and he had no light perception (NLP; OS). There was a relative afferent pupillary defect (RAPD) in his left eye. Fundus examination demonstrated pallor in the bilateral temporal part of the optic disc. A visual field examination was not performed because of his bad VA.

**Figure 1:**
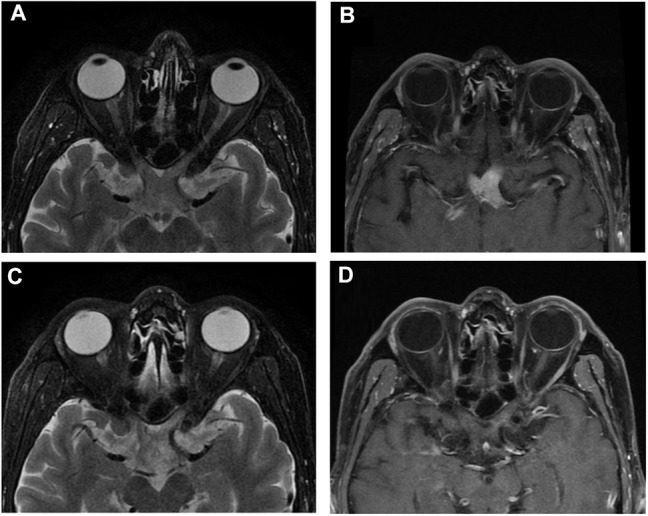
Orbital MRI of Case 1. **(A,B)** Orbital MRI showed chiasm enlargement in T2-weight imaging and its intrinsic homogenous enhancement of the optic chiasm in T1-weight enhanced imaging when the patient first visited the clinic. **(C,D)** MRI showed moderate enlargement in T2-weight imaging with a light intrinsic enhancement in T1-weight imaging of the optic chiasm after steroid treatment.

Results of a complete blood count (CBC), erythrocyte sedimentation (ESR), C-reactive protein (CRP), and lymphocyte subpopulation were normal, and HIV titer was negative. A cerebrospinal fluid (CSF) test showed a normal white blood cell (WBC) count (3 × 10^6^/L; normal range: <10 × 10^6^/L) with several leukomonocytes, elevated protein levels (721 mg/L; normal range: 150–400 mg/L), and immune abnormalities. CSF immunoglobulin A (IgA) was 0.684 mg/dl (normal range: 0–0.5 mg/dl), IgG was 4.35 mg/dl (normal range: 0–3.4 mg/dl), and IgM was 0.21 mg/dl (normal range: 0–0.13 mg/dl). Orbit MRI revealed thickening of the optic chiasma in T1- and T2-weighted imaging and significant homogenous enhancement with gadolinium (Figures 1A,B). Brain MRI did not show any other lesions.

The patient was suspected of optic neuritis and administered intravenous steroids (1 g of methylprednisolone once daily for 3 days, and sequential taper). His right eye's VA was improved from 0.02 to 0.5, 1.5 months later, but the left eye remained NLP. The visual field of his right eye demonstrated a dense temporal hemianopic defect in remission.

Four months after his first presentation, the patient came back to the neuro-ophthalmology clinic, reporting fatigue, somnolence, aches in his back and all four extremities, slow speaking, and unstable walking. His right VA had deteriorated to 0.25, while his left VA remained NLP. His blood test result was unremarkable. There was a significant increase in CSF WBC count (35 × 10^6^/L) and protein (2168.4 mg/L) and immunoglobulin levels (IgA 1.95 mg/dl, IgG 9.08 mg/dl, IgM 2.06 mg/dl). Orbit MRI showed that the bulky optic nerve had become thinner and less intense than in the prior examination (Figures 1C,D). Meanwhile, multiple lesions in the periventricular area, the lenticular nucleus, and the thalamus were observed in T1- and T2-weighted imaging and enhanced with gadolinium in brain MRI.

**Figure 2:**
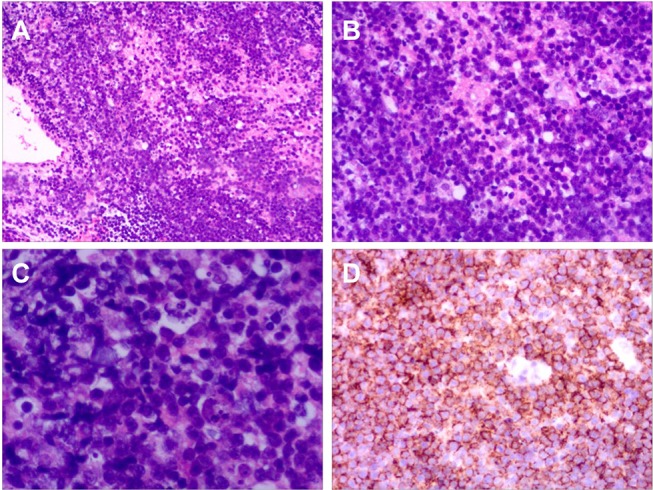
Histopathology of Case 1. **(A)** Haematoxylin and eosin (H&E) ×10. **(B)** H&E ×25. **(C)** H&E ×100. H&E staining for brain tissue biopsy showed diffuse proliferation of large atypical lymphoid cells. **(D)** Immunohistochemical staining for CD20 showed that most cells were positive.

A brain biopsy was conducted on tissue obtained via a frontal approach. Histopathology of the specimen showed diffuse proliferation of large atypical lymphoid cells (Figure 2). Immunohistochemical staining was positive for CD20 in most lymphocytes and CD3 in scattered lymphocytes. A bone marrow biopsy demonstrated no malignant cells. Although there is no pathologic examination of the chiasm and optic nerve, it is reasonable to assume that the pathological brain process is the same for both. The final diagnosis was primary central nervous system diffuse large B-cell lymphoma with optic nerve and chiasm infiltration.

The patient underwent three courses of treatment with high doses of methylprednisolone and methotrexate plus rituximab, which resulted in a moderate improvement of his central nervous system (CNS) symptoms but not of his VA in either eye. One month later, his condition deteriorated, and he died.

### Case 2

A 67-year-old woman presented to the neuro-ophthalmology clinic of the PLAGH in December 2015 with bilateral transient visual obscuration for 3 months. She did not report headache, weight loss, or other constitutional symptoms but referred to night sweats.

**Figure 3:**
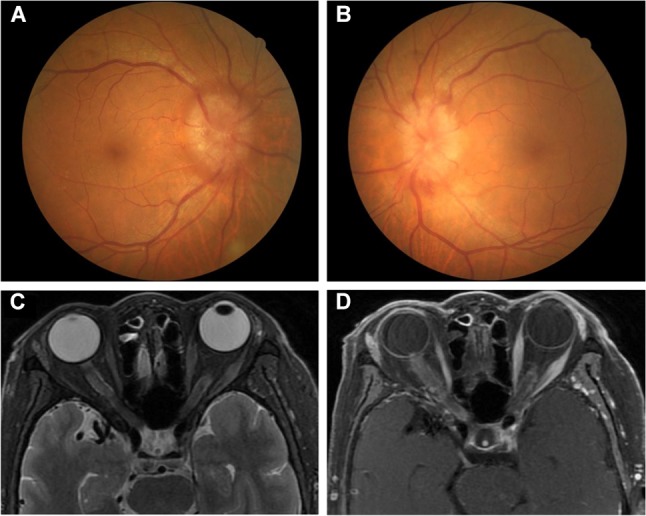
**(A,B)** Fundus examination in Case 2. Bilateral optic disc oedema. **(C,D)** Orbital MRI in Case 2. **(C)** Bilateral optic nerve enlargements of orbital and inner canal segments in T2-weight imaging. **(D)** Bilateral leptomeningeal enhancement in optic nerves when gadolinium was administered (“tram-tracking” sign).

On admission, her best-corrected VA was 0.8 in both eyes. There was no RAPD in either eye. In a slit lamp examination, the anterior segments of both eyes were unremarkable. The vitreous body was clear. Fundus examination demonstrated optic disc swelling bilaterally (Figures 3A,B). Bilateral visual fields were tubular.

A CBC showed a high WBC (12.15 × 109/L) with a low neutrophilic granulocyte percentage (31.6%) and a high lymphocyte percentage (59.1%). ESR was 87 mm/h. Lymphocyte subpopulation revealed that the B lymphocyte rate was markedly elevated (0.64; normal range: 0.05–0.18), while the percentages of CD3 (30%; normal range: 58–84%), CD4 (18%; normal range: 27–50%), and CD8 (12%; normal range: 19–42%) T lymphocytes and NK cells (4%; normal range: 7–40%) were decreased. The results of other blood examinations were unremarkable, and HIV titer was negative. A CSF test showed a normal WBC count (6 × 106/L) with several leukocytes, high protein levels (1057.2 mg/L), and immune abnormalities (IgA 0.901 mg/dl, IgG 12.5 mg/dl, IgM 45.5 mg/dl). Orbit MRI demonstrated optic nerve enlargements with high T2 signal involving both the orbit and the inner canal segment and mild sheath enhancement after gadolinium administration (“tram tracking” sign) (Figures 3C,D). Ultrasound imaging of the neck, armpits, and groins did not reveal abnormal lymph nodes.

A bone marrow biopsy revealed a low-grade lymphoproliferative disorder, and Gomori staining was positive. Bone marrow flow cytometry with lymphocyte markers was positive for B cell markers CD19, CD20, CD22, and cytoplasm lambda light chains, which suggested B cell clone malignant neoplasms (small B cell type). The final diagnosis was metastatic optic neuropathy of small B cell non-Hodgkin lymphoma. The patient refused treatment for unknown reasons and was discharged without follow-up.

## Literature Review

Optic nerve lymphoma can be divided into four categories: isolated optic nerve involvement, optic nerve involvement with central nervous system (CNS) disease, optic nerve involvement with systemic disease, and optic nerve involvement with primary intraocular lymphoma (5), which may allow for a better understanding of the origin of the disease.

Although a large number of intraocular lymphoma cases have been reported, these and other complicated cases were excluded from this review, as its primary purpose was to identify characteristics of optic nerve lymphoma. Moreover, although several cases were reported before 1990, given the limited use of MRI at that time, only cases between 1990 to 2018 were included. We examined 22 immunocompetent cases in the literature, including the two cases reported in this paper, presenting lymphomatous infiltration of the optic nerves with pathological evidence (Table 1). The patients were aged between 21 and 87 years (median age: 66.5 years). Eleven of them were male. Twelve patients suffered bilaterally, and 10 suffered unilaterally. Thirteen cases were confirmed by optic nerve biopsy, and the others were confirmed by extra–optic nerve biopsy. Thirteen cases were classified as optic nerve involvement with systemic disease, five as isolated optic nerve involvement, and four as optic nerve involvement with PCNSL. The initial symptom in 21 patients was vision deterioration, and four of them simultaneously presented visual field defects. One patient (Case 14) complained about visual field defects only (4). Besides visual impairment and visual field defects, one patient complained about flashing lights on his first visit as well (6). Fourteen patients exhibited optic disc swelling in their affected eyes when they were first admitted to the ophthalmology clinic, three exhibited pallor, one had a normal optic disc, while no relevant information was provided in three cases.

**Table 1:** Cases of optic nerve lymphoma.

**Patients' number**	**References**	**Age/ sex**	**Diagnosis**	**Method of diagnosis**	**Classification**	**Initial complaint**	**Optic disc at present**	**Lumbar puncture**	**MRI**				**Treatment**	**VA outcome**
									**Enlargement**	**Enhancement**	**Intrinsic/ sheath**	**Location**		
1	(6)	44/M	Hodgkin's disease	Chiasm and lymph node biopsy	PCNSL	Decreased VA and flashing lights (OU)	Pallor (OU)	n/a	Yes	n/a	n/a	Optic chiasm	Radiotherapy, chemotherapy and corticosteroid	n/a (died)
2	(7)	21/M	Hodgkin's disease	Lymph node biopsy	Systemic lymphoma	Decreased VA (OS)	Edema (OS)	wnl	Yes	Marked	Intrinsic and sheath enhancement	Left intraorbital and intracanal optic nerve	Radiotherapy and corticosteroid	Improved
3	(8)	42/F	NHL	Lymph node biopsy	Systemic lymphoma	Decreased VA (OD) VF defect (OS)	Pallor (OU)	High protein	Yes	n/a	n/a	Optic chiasm	Radiotherapy	Stable
4	(9)	69/F	Small cleaved-cell lymphoma	Temporal artery biopsy and bone marrow biopsy	Systemic lymphoma	Decreased VA (OS)	Normal (OS)	High protein	Yes	Marked	Intrinsic and sheath enhancement	Left intraorbital, intracanal optic nerve and anterior chiasm	Radiotherapy and corticosteroid	Improved
5	(10)	74/F	B-cell NHL	Optic nerve biopsy	Systemic lymphoma	Transient decreased VA and VF defect (OS)	Edema (OU)	High protein	Yes	No	n/a	Optic nerve^*^	Radiotherapy	Improved
6	(11)	87/F	B-cell NHL	Lymph node and bone marrow biopsy	Systemic lymphoma	Decreased VA (OU)	Edema (OU)	n/a	Yes	Marked	Intrinsic and sheath enhancement	Bilateral intraorbital optic nerve	Corticosteroid and chlorambucil	Improved
7	(12)	70/F	NHL	Bone marrow biopsy	Systemic lymphoma	Decreased VA (OU)	Mild edema (OU)	wnl	Yes	Yes	Intrinsic and sheath enhancement	Bilateral optic nerves^*^	Corticosteroid and chlorambucil	Improved
8	(2)	66/F	B-cell lymphoma	Optic nerve and sheath biopsy	Optic nerve lymphoma	Decreased VA (OD)	Edema (OD)	wnl	n/a	Yes	n/a	Right intraorbital and intracanal optic nerve^*^	Corticosteroid	Deteriorated
													Radiotherapy and chemotherapy	Stable
9	(13)	49/M	Small B-cell lymphoma	Optic nerve and sheath biopsy	Systemic lymphoma	Decreased VA and VF defect (OS)	Edema (OS)	n/a	Yes	Moderate	Intrinsic and sheath enhancement	Left intraorbital and intracanal optic nerve	Radiotherapy	Stable
10	(14)	69/M	Small B-cell NHL	Optic nerve and sheath biopsy	Optic nerve lymphoma	Decreased VA (OU)	Edema (OS)	n/a	Yes	n/a	n/a	Bilateral intraorbital optic nerve and chiasm	Radiotherapy	Stable
11	(15)	39/M	T-cell NHL	Optic nerve biopsy	Systemic lymphoma	Decreased VA (OU)	Edema (OU)	n/a	n/a	Mild	Intrinsic and sheath enhancement	Right optic nerve and chiasm^*^	Radiotherapy and chemotherapy	Transient improved
12	(3)	72/M	Small B-cell NHL	Optic nerve biopsy	PCNSL	Decreased VA (OD)	Pallor (OD)	n/a	Yes	Marked	Intrinsic enhancement	Optic chiasm, tract and lateral geniculate	Chemotherapy	n/a (died)
13	(16)	44/M	B-cell NHL	Retroperitoneal mass biopsy	Systemic lymphoma	Decreased VA (OS)	Mild edema (OS)	High protein and B-cell neoplasm	Yes	Mild	Intrinsic and soft tissue along the optic nerve enhancement	Intraorbital optic nerve	Corticosteroids radiotherapy and chemotherapy	Stable
14	(4)	59/M	Large B-cell NHL	Optic nerve (optic tract)	Optic nerve lymphoma	VF defect (OU)	n/a	n/a	Yes	Marked	Intrinsic enhancement	Optic nerve, chiasm and tract	Chemotherapy	Mild improved
15	(17)^#^	84/F	Large B-cell NHL	Optic nerve biopsy	Optic nerve lymphoma	Decreased VA (OU)	n/a	n/a	Yes	Marked	Intrinsic enhancement	Optic chiasm	Corticosteroids	n/a
16	(17)^#^	67/M	Large B-cell NHL	Optic nerve biopsy	PCNSL	Decreased VA (OD)	Edema (OD)	n/a	Yes	Marked	Intrinsic enhancement	Optic canal and chiasm	Corticosteroids	Transient improved
17	(5)^$^	65/F	B-cell NHL	Optic nerve sheath biopsy	Systemic lymphoma	Decreased VA (OD)	Edema (OD)	n/a	Yes	Moderate	Sheath enhancement	Intraorbital optic nerve	Radiotherapy	NLP
18	(5)^$^	25/F	Burkitt lymphoma	Optic nerve and sheath biopsy	Systemic lymphoma	Decreased VA (OS)	Edema (OD)	n/a	Yes	Marked	Intrinsic and soft tissue along the optic nerve enhancement	Left intraorbital optic nerve	Radiotherapy	n/a (died)
19	(18)	67/M	NHL	Post-mortem of the optic nerve	Systemic lymphoma	Decreased VA (OU)	Edema (OU)	wnl	Yes	Marked	Intrinsic and sheath enhancement	Right intraorbital optic nerve	Corticosteroids and intrathecal methotrexate	Improved
20	(19)	68/F	B-cell NHL	Optic nerve biopsy	Optic nerve lymphoma	Decreased VA (OU)	n/a	n/a	Yes	Marked	Intrinsic enhancement	Bilateral optic nerve, chiasm and tract	Corticosteroids radiotherapy and chemotherapy	n/a
21	Case 1	55/M	Large B-cell NHL	Brain biopsy	PCNSL	Decreased VA (OU)	Mild edema (OD) and pallor (OS)	High protein	Yes	Marked	Intrinsic enhancement	Optic chiasm and tract	Corticosteroids	Improved
22	Case 2	67/F	Small B-cell NHL	Bone marrow biopsy	Systemic lymphoma	Decreased VA (OU) VF defect (OU)	Edema (OU)	High protein	Yes	Moderate	Intrinsic and sheath enhancement	Bilateral intraorbital optic nerve	No	n/a

*M, male; F, female; NHL, non-Hodgkin lymphoma; OD, right eye; OS, left eye; OU, both eyes; VA, visual acuity; VF, visual field; NLP, no light perception; n/a, information not available; CBC, complete blood count; wnl, within normal limits*.

* *MRI images were not provided*.

# *Case 15 and 16 are different patients from the same study*.

$ *Case 17 and 18 are different patients from the same study*.

Significant enlargement of the lesions was observed in orbital/brain MRI in nearly all patients, except for two whose optic nerves were not described by the authors or MR images were not provided in the respective reports (2, 10). Most lesions were located at the intraorbital and intracanal optic nerves and the chiasm; only three cases showed optic tract involvement. Eighteen cases were examined by orbital/brain MRI with gadolinium. Seventeen of them showed abnormal contrast enhancement of the anterior visual pathway, while one patient, whose MR images were not provided in the report, was described without enhancement by Dayan et al. (10). Of the 17 enhanced cases, 11 showed marked enhancement, 3 moderate, and 2 subtle, while intensity was not described in 2 reports. Significant enhancement was observed in all the PCNSL and isolated optic nerve lymphoma patients whose images were described by the authors. However, moderate and subtle enhancement was found in the systemic lymphoma metastatic to the optic nerve only. According to enhanced MRI, we found that lesions could be characteristic of optic nerve intrinsic involvement, sheath and orbital soft tissue involvement, or both. In five cases, enhanced MRI was not described, six were intrinsic involvement, one was sheath involvement, and ten were both. Of the five isolated optic nerve lymphoma, four underwent enhanced MRIs. Three out of four demonstrated intrinsic enhancement, while in one case, the site of the lesion was not described and MRI images were not provided. Enhanced MRI was provided for all four PCNSL patients; three of them demonstrated intrinsic enhancement and one showed no enhancement. MRIs from 13 systemic disease cases were complicated. Ten of them demonstrated both intrinsic and sheath and soft tissue enhancement after administration of gadopentetic acid, one showed leptomeninges enhancement, and one showed no enhancement.

Cerebrospinal fluid data were available in ten cases. Six patients had elevated CSF protein, and only one had a neoplasm. We found abnormality of CSF immunity in both of our cases, which may play a role in detecting optic nerve lymphoma.

Radiotherapy, chemotherapy, and corticosteroids were the most common treatments for optic nerve lymphoma. After treatment, most patients' VA was stable, improved, or transiently improved. It is worth noting that the visual acuity of Case 8 decreased after an initial treatment with steroids but was stabilized after radiotherapy combined with chemotherapy.

## Discussion

Early diagnosis of optic nerve lymphoma is essential in the management of these cases if vision is to be preserved (8). However, it is challenging due to the condition's similarity to a variety of inflammatory diseases and neoplastic infiltrations in clinical and radiological examinations (20). Optic nerve biopsy can lead to the diagnosis, but always carries a risk of permanent visual loss (2) and may yield false-negative results since the size of the biopsy specimen is limited by the necessity of preserving vision (10). Matsuyama et al. reported an enigmatic case initially misdiagnosed as inflammatory infiltration (17), as was Case 1 reported in this paper. Therefore, it is essential to find some flags for diagnosing anterior visual pathway involvement of lymphoma.

Brain or orbit MRI always plays an essential role in the diagnosis and differentiation of optic neuropathies (5). Enlargement of the lesion is an important MRI finding in neoplastic disorders, including lymphomatous infiltration of the optic nerve (21, 22). Even though optic nerve enlargement could also be seen in inflammatory optic neuritis, the lesion showed 10% or less thicken compared to the normal (23), and the inflammation reaction usually lasts a few weeks and then diminishes. In this review, all lesions were found abnormally large in MRI. Kim et al. (5) reported an elderly woman with an affected optic nerve and sheath swelling to 7 mm compared to the contralateral optic nerve, which was only 3 mm in diameter. Although the authors thought it was consistent with optic neuritis, we considered that the lesion was too large to consider an inflammatory disease. Siatkowski et al. examined an affected optic nerve using B-scan and found that its diameter was 6.3 mm, which led them to suspect lymphoma infiltration (7). Ahle et al. described optic nerve infiltration in 7 patients from a 752–PCNSL cohort; MRI showed optic nerve thickening in only three of the seven cases. However, the authors considered that standard MRI findings without sequences focused on the optic nerves might be interpreted as usual, and that orbital MRI focusing on both optic nerves and including high-resolution fat-suppressed sequences might increase the chance of detecting optic nerve enlargement (24). Optic nerve lymphoma usually manifests as massive nerve swelling (2, 18) and could last for a long time without treatment. According to MRI, almost all bulky lesions in our review affected the intraorbital or intracanal segment of the optic nerve, which may lead to increased optic nerve sheath pressure, resulting in severe optic disc oedema. Chiasm and intracranial portion involvement always manifested as pallor, regular, and sometimes mild oedema, which may be explained by the lack of optic nerve sheath.

Contrast enhancement was seen in MRI in most optic nerve lymphoma patients. According to Ahle et al. (24), MRI showed contrast enhancement of the optic nerve in all seven optic nerve infiltrated patients described. In our review, enhanced MRI was observed in 19 of the 22 cases. Only one case, whose MR images were not provided in the report, was not described as contrast enhancement (10). Wong et al. (18) reported a patient whose enhanced MRI was described as regular during the administration; however, enhancement was found during the progression of the disease. Therefore, when the enhanced MRI scan of a suspected optic nerve lymphoma patient was normal, a repeat examination during the progression of the disease was needed. This may provide useful information.

Enhanced MRI was also crucial in speculating the spread of the disease. Given that isolated optic nerve lymphoma is always characterized by malignant lymphoma cell infiltration of the optic nerve, PCNSL often involves brain parenchyma, and lymphoma metastatic to the CNS is usually found in the leptomeninges or dura (25), the site and intensity of the lesions from enhanced MRI was supposed to be helpful in differentiating the primary cause of optic nerve lymphoma. In this review, nearly all isolated optic nerve lymphoma and PCNSL patients showed marked homogenous intrinsic enhancement. Although most systemic lymphoma cases were characterized by significant enhancement with both nerve sheath, leptomeninges, or soft tissue along the optic nerve and optic nerve involvement, some of them demonstrated moderate or subtle enhancement with both or only sheath involvement. These findings suggest that isolated optic nerve lymphoma and PCNSL often involve the optic nerve fiber bundles (intrinsic), while metastatic optic nerve lymphoma tends to invade the optic nerve sheath and soft tissue along the optic nerve first, and subsequently diffuses to optic nerve fiber bundles. Kitzmann et al. (15) and Matsuyama et al. (17) did not find malignant lymphocytes in the sheath of their metastatic patients whose MRI showed sheath enhancement, which may be due to insufficient tissue collected or steroid use before biopsy (2).

Kim et al. (5) described imaging characteristics of pathologically identified optic nerve lymphoma patients and analyzed the mechanism of the spread in their literature, however, it mainly focused on the relationship between the pathogenesis and histology. Herein, we further focused on the relationship between the spread of the disease and the location of the abnormal signal in enhanced MRI (intrinsic, leptomeningeal, or both), and tried to tell that enhanced MRI may help to point the primary cause of optic nerve lymphoma. What needs to be emphasized is that it is challenging to differentiate optic nerve lymphoma from other neoplastic lesions using enhanced MRI only. Variable enhancement may also be seen in the setting of optic nerve glioma, optic nerve sheath meningioma, inflammatory pseudotumor, tumor seeding, and so on (26). It only leads to suspicion, and needs additional works if necessary.

CSF analysis is another vital factor in the diagnosis of optic nerve lymphoma. At the time of diagnosis, at least one of the routine CSF indices is abnormal in more than 80% of CNS lymphomas (27). Series that have tested CSF in a variety of lymphomas with CNS involvement showed that CSF cell counts were normal in 33–60% of patients, and protein was normal in 33–55% of patients (28). Scott et al. considered CSF cytology the gold standard in the diagnosis of CNS lymphoma due to its high specificity (≥95%) despite its relatively low sensitivity (<50%) (28). Only one patient included in this review had tumor cells detected. A possible reason for this might be an insufficient amount of CSF samples, which should be >10.5 ml (28). High CSF protein levels were observed in most patients in the series, including our two patients despite its low specificity. We found a significant elevation of CSF protein in Case 1 during the progression of the disease. Although some investigators reported that CSF protein abnormalities did not reliably predict CSF involvement of lymphoma (29, 30), we suspected that extremely high levels of protein might be correlated with lymphoma involvement of the CNS (including optic nerves). Few studies have evaluated immunoglobulin in patients with suspected anterior visual pathway involvement of PCNSL or systemic lymphoma. Examining CSF immunoglobulin in our two patients, we found elevated IgA, IgM, and IgG in both. Therefore, we recommend paying close attention to CSF immunoglobulin in suspected optic nerve lymphomatous infiltration.

There are two limitations in our study, the first one is the small number of patients. Optic nerve lymphoma is a rare disease, and cases were limited in most reports. However, it is valuable to study more patients in the future to draw clear conclusions if it possible. The other one is indirect biopsy evidences to support the final diagnosis. Although direct histopathology provided confirmation of optic nerve lymphoma diagnosis, it should be judiciously recommended due to its risk of permanent visual loss (2). Thirty-six percent of the cases in this series were confirmed by extra–optic nerve biopsy, as were our two cases. Although there is no pathological examination of the optic nerve in these cases, it is reasonable to assume that they are single lymphomas involving optic nerves rather than multiple diseases affecting the optic nerve. Other than optic nerve biopsy, Ahle et al. diagnosed optic nerve lymphoma in their patients by MRI and CSF analysis (24).

## Conclusion

In clinical practice, in the absence of specific clinical features of optic nerve lymphoma, a combination of available radiographic features and diagnostic CSF tests may be used to establish the suspicion quickly and at the lowest risk for the patient. Considerable enlargement and different patterns of enhancement of optic nerves in orbit MRI and significant elevation of CSF protein and immunoglobulin levels may suggest anterior visual pathway involvement of lymphoma. However, it is of limited value because there are only two newly diagnosed patients added into the literature, studies included more patients should be performed in the future.

## Data Availability Statement

All datasets generated for this study are included in the article/supplementary material.

## Ethics Statement

This study was carried out in accordance with the recommendations of the Human and Research Ethics Committees of the First Medical Center, Chinese PLA General Hospital, Beijing, China. The protocol was approved by the Human and Research Ethics Committees of the First Medical Center, Chinese PLA General Hospital. All subjects gave written informed consent in accordance with the Declaration of Helsinki. Written informed consent was obtained from the patient or his next of kin for the publication of any potentially identifiable images or data included in this article.

## Author Contributions

MY: conceptualization, methodology, data curation, and writing—original draft. JZ: resources and supervision. SW: investigation, supervision, and project administration. HS: writing—original draft. HZ: resources and visualized the original work. QX: resources, writing—review and editing, and supervision.

## Conflict of Interest

The authors declare that the research was conducted in the absence of any commercial or financial relationships that could be construed as a potential conflict of interest.

## Abbreviations

PCNSL: primary central nervous system lymphoma
MRI: magnetic resonance imaging
VA: visual acuity
NLP: no light perception
RAPD: relative afferent pupillary defect
CBC: complete blood count
ESR: erythrocyte sedimentation
CRP: C-reactive protein
CSF: cerebrospinal fluid
WBC: white blood cell count
CNS: central nervous system.
